# An fMRI study of affective perspective taking in individuals with psychopathy: imagining another in pain does not evoke empathy

**DOI:** 10.3389/fnhum.2013.00489

**Published:** 2013-09-24

**Authors:** Jean Decety, Chenyi Chen, Carla Harenski, Kent A. Kiehl

**Affiliations:** ^1^Department of Psychology, University of Chicago, ChicagoIL, USA; ^2^Department of Psychiatry and Behavioral Neuroscience, University of Chicago, ChicagoIL, USA; ^3^Departments of Psychology and Neuroscience, University of New Mexico, AlbuquerqueNM, USA; ^4^Mind Research Network, AlbuquerqueNM, USA

**Keywords:** amygdala, effective connectivity, empathy, insula, orbitofrontal cortex, perspective taking, psychopathy, ventral striatum

## Abstract

While it is well established that individuals with psychopathy have a marked deficit in affective arousal, emotional empathy, and caring for the well-being of others, the extent to which perspective taking can elicit an emotional response has not yet been studied despite its potential application in rehabilitation. In healthy individuals, affective perspective taking has proven to be an effective means to elicit empathy and concern for others. To examine neural responses in individuals who vary in psychopathy during affective perspective taking, 121 incarcerated males, classified as high (*n* = 37; Hare psychopathy checklist-revised, PCL-R ≥ 30), intermediate (*n* = 44; PCL-R between 21 and 29), and low (*n* = 40; PCL-R ≤ 20) psychopaths, were scanned while viewing stimuli depicting bodily injuries and adopting an imagine-self and an imagine-other perspective. During the imagine-self perspective, participants with high psychopathy showed a typical response within the network involved in empathy for pain, including the anterior insula (aINS), anterior midcingulate cortex (aMCC), supplementary motor area (SMA), inferior frontal gyrus (IFG), somatosensory cortex, and right amygdala. Conversely, during the imagine-other perspective, psychopaths exhibited an atypical pattern of brain activation and effective connectivity seeded in the anterior insula and amygdala with the orbitofrontal cortex (OFC) and ventromedial prefrontal cortex (vmPFC). The response in the amygdala and insula was inversely correlated with PCL-R Factor 1 (interpersonal/affective) during the imagine-other perspective. In high psychopaths, scores on PCL-R Factor 1 predicted the neural response in ventral striatum when imagining others in pain. These patterns of brain activation and effective connectivity associated with differential perspective-taking provide a better understanding of empathy dysfunction in psychopathy, and have the potential to inform intervention programs for this complex clinical problem.

Empathy, the social-emotional response that is induced by the perception of another person's affective state, is a fundamental component of emotional experience, and plays a vital role in social interaction (Szalavitz and Perry, 2010). It is thought to be a proxy for prosocial behavior, guiding our social preferences and providing the affective and motivational base for moral development. Empathy is a deeply fundamental component of healthy co-existence whose absence is the hallmark of serious social-cognitive dysfunctions. Among the various psychopathologies marked by such deficits, psychopaths are characterized by a general lack of empathy and attenuated responding to emotional stimuli (Blair et al., 1997; Herpertz and Sass, 2000; Hare, 2003; Mahmut et al., 2008).

Empathy includes both cognitive and affective components (Decety and Jackson, 2004; Shamay-Tsoory, 2009; Singer and Lamm, 2009; Decety, 2011a; Zaki and Ochsner, 2012). The empathic arousal component, or emotion contagion, develops earlier than the cognitive component, and seems to be hardwired in the brain with deep evolutionary roots (Decety and Svetlova, 2012). In addition developmental research has found that concern for others emerges prior to the second year of life. In these studies, young children are not only moved by others' emotional states, but they make distress and pain attribution in conjunction with their comforting behavior and recognize what the target is distressed about (Roth-Hanania et al., 2011). Empathic arousal plays a fundamental role in generating the motivation to care and help another person in distress and depends only minimally on mindreading and perspective-taking capacities. In naturalistic studies, young children with high empathy disposition are more readily aroused vicariously by other' sadness, pain or distress, but at the same time possess greater capacities for emotion regulation so that their own negative arousal motivates rather than overwhelms their desire to alleviate the other's distress (Miller and Jansen op de Haar, 1997; Nichols et al., 2009). Empathic arousal is a bottom-up process in which the amygdala, hypothalamus, anterior insula (aINS), and orbitofrontal cortex (OFC) underlie rapid and prioritized processing of emotion signals sent by others (Decety and Svetlova, 2012). The cognitive component of empathy overlaps with the construct of perspective taking (Ruby and Decety, 2003). Perspective taking describes the ability to consciously put oneself into the mind of another individual and imagine what that person is thinking or feeling. The ability to adopt the perspective of another has previously been linked to social competence and social reasoning (Underwood and Moore, 1982). A substantial body of behavioral studies has documented that affective perspective taking is a powerful way to elicit empathy and concern for others (Batson et al., 1997; Decety and Hodges, 2006; Van Lange, 2008). For instance, Oswald (1996) found that affective perspective taking is more effective that cognitive perspective taking to evoke empathy and altruistic helping. Functional neuroimaging studies have consistently identified a circumscribed neural network reliably involved in perspective taking, which links the medial prefrontal cortex (mPFC), posterior superior temporal sulcus (pSTS/TPJ), and temporal poles/amygdala (Ruby and Decety, 2003, 2004; Hynes et al., 2006; Lawrence et al., 2006; Vollm et al., 2006; Rameson et al., 2011). Lesion studies have shown that affective perspective taking depends on intact medial and ventromedial prefrontal cortex (vmPFC) as well as regions in the posterior temporo-parietal cortex (Rankin et al., 2006). Importantly, neurological patients with damage to the vmPFC are found to exhibit a specific impairment in affective theory of mind tasks, sparing their cognitive empathy ability (Shamay-Tsoory et al., 2006).

In the empathy literature, a number of behavioral studies have documented a distinction between an imagine-self perspective and an imagine-other perspective (Batson, 2011). When adopting the former perspective, the central figure is oneself and one's own thoughts and feelings, and increases the salience of self-attributes. The imagining-other perspective involves an empathic attentional set in which the individual opens himself or herself in a deeply responsive way to the other person (Barrett-Lennard, 1981; Batson, 2009; Halpern, 2012). This distinction between imagine-self and imagine-other perspectives is also supported by functional neuroimaging research. For instance, when participants are asked to imagine being in physical pain themselves, they report greater pain intensity ratings and have greater activation in the aINS, aMCC, thalamus, and somatosensory cortex compared to imagining the same pain happening to another person (Jackson et al., 2006). The reverse contrast, imagining-other in pain vs. imagining oneself in pain, was associated with increased activity in the right pSTS and mPFC. Another study reported that self-perspective compared to other-perspective, when watching videos depicting facial expression of pain, led to higher activity in brain areas involved in the affective response to threat or pain, such as the amygdala, the insula, and the aMCC, as well as higher subjective ratings of personal distress (Lamm et al., 2007).

It is well established that individuals with psychopathy have limited aversive arousal to the distress and sadness cues of others (Van Honk and Schutter, 2006; Blair, 2007; Anderson and Kiehl, 2011), but spared theory of mind and cognitive perspective taking capacities (Blair, 2005; but see Brook and Kosson, 2012). However, it is not known if, when they adopt the affective perspective taking of another person, the extent to which the active contemplation of another's affective experience modulates brain circuits involved in affective processing.

Building on past research on perspective taking and empathy with healthy participants (Jackson et al., 2006; Lamm et al., 2007; Decety and Porges, 2011) as well as a recent study of pain empathy in criminal psychopaths (Decety et al., 2013), incarcerated offenders with different levels of psychopathy on Factors 1 and 2 underwent fMRI scanning while watching visual stimuli depicting physical pain. To elicit first- or third-person perspective taking (or imagine-self and imagine-other perspectives respectively) we explicitly manipulated the task instructions given to the participants in the scanner before each block, by asking them to think of the situations as either occurring to them or to someone else. Factor 1 describes a constellation of affective and interpersonal traits considered to be fundamental to the construct of psychopathy, which includes shallow affect, callous and lack of empathy, while Factor 2 reflects an unstable and antisocial lifestyle (Hare, 2003). Based on fMRI studies that used similar instructions and stimuli with healthy participants, it was predicted that imagine-self perspective would be associated with stronger visceromotor response in the aINS, somatosensory cortex and ACC than imagine-other perspective taking in participants scoring low on the psychopathy checklist-revised (PCL-R), especially Factor 1, because these regions have been associated with activation of representations of pain and of other negative emotions (Benuzzi et al., 2008). However, due to altered responding to affective stimuli in psychopathy, the opposite effect was expected for individuals scoring high on psychopathy PCL-R Factor 1. When instructed to adopt the perspective of another individual in physical pain, we hypothesized that individuals scoring high on the PCL-R would show a pronounced deficit in aINS and vmPFC hemodynamic response. This prediction is based on the large body of evidence from lesion studies and neuroimaging studies with healthy individuals as well as with psychopaths that show the importance of these regions in affective perspective taking and empathic concern (Rankin et al., 2003; Shamay-Tsoory et al., 2003; Kiehl, 2006; Gleichgerrcht et al., 2011; Rameson et al., 2011; Decety et al., 2012; Young and Dungan, 2012). The distinction between imagine-self and imagine-other is critical, as most studies suggest that psychopaths have spared mentalizing (cognitive empathy) abilities, and that the key deficit appears to relate to their lack of concern about the impact of their behavior on potential victims, rather than the inability to adopt a victim-centered perspective (Dolan and Fullam, 2004).

Finally, analyses of functional segregation can be complemented by effective connectivity analyses. Whereas standard contrast analyses create a “snapshot” of regional brain activity in response to a task or condition, functional connectivity analyses can identify patterns of communication between regions that contrast analyses may not detect [see Decety and Porges, 2011; Zaki et al. (2007) for such methods in empathy for pain]. Given the role of the insula in mapping internal states of bodily and subjective feelings (Craig, 2002) and that of the amygdala in motivational salience (Cunningham and Brosch, 2012), these two regions were selected as seeds for the functional connectivity analyses.

## Materials and methods

### Participants

One hundred twenty-four adult right-handed males between the ages of 18 and 50, incarcerated in a medium-security North American correctional facility, volunteered for the study and provided informed consent to the procedures described here, which were approved by the Institutional Review Boards of the University of New Mexico and the University of Chicago. Participants underwent the PCL-R assessment, including file review and interview, conducted by trained research assistants under the supervision of Dr. Kiehl. Three participants were excluded for excessive movement in the scanner. Participants scoring 30 and above on the PCL-R were assigned to the high-psychopathy group (*n* = 37; age 32.5 ± 7.8; IQ 103.3 ± 13). To create the medium- and low-psychopathy groups, two groups of volunteers were matched to high scorers on age, race and ethnicity, IQ (WAIS), comorbidity for DSM-IV Axis II disorders, and past drug abuse and dependence, from pools of incarcerated volunteers scoring between 21 and 29 (*n* = 44; age 34.1 ± 7; IQ 97.3 ± 12.7), and volunteers scoring below 20 on the PCL-R (*n* = 40; age 34.6 ± 6.9; IQ 99.3 ± 14), respectively. Participants were paid for their participation in the study.

### Exclusion criteria

Additional participants who volunteered for the study but met exclusion criteria were not included. Exclusion criteria were age younger than 18 years or older than 55, non-fluency in English, reading level lower than 4th grade, IQ score lower than 80, history of seizures, prior head injury with loss of consciousness > 30 min, current Diagnostic and Statistical Manual of Mental Disorders (4th ed.; American Psychiatric Association, 1994) Axis I diagnosis, lifetime history of a psychotic disorder or psychotic disorder in a first degree relative, or current alcohol or drug use.

### Task design

Participants in the MRI scanner were instructed to adopt either a self-perspective or an other-perspective while viewing visual stimuli depicting right hands and right feet of individuals in painful and non-painful situations [stimuli and procedure similar to Jackson et al. (2006)]. All stimuli showed familiar events that can happen in everyday life to people (e.g., pinching one's finger in a door, or catching one's toe under a heavy object). Various types (mechanical, thermal and pressure) of pain inflicted to the limbs were depicted. Neutral pictures showed limbs in visually similar situations without pain component (e.g., a hand on the handle of a drawer as opposed to being caught in the same drawer). Participants viewed 120 stimuli of pain and no pain. Each trial lasted 1.4 s and consisted of one of the pain scenarios, and the inter-stimuli intervals were jittered between 2.5 and 5.4 s. Timing parameters were generated using a genetic optimization algorithm (Wager and Nichols, 2003). Eye-tracking was monitored in the scanner to ensure that participants were paying attention to the stimuli.

### Perspective instructions

A mixed block-event related fMRI design [24 blocks (12 imagine-self and 12 imagine-other) with a total 120 trials] was employed, in which instructions were given to the subjects at the beginning of each block, i.e., for the imagine-self perspective blocks (“*Imagine that these situations are happening to you*”), and for the imaging-other perspective blocks (“*Imagine that these situations are happening to someone else*”). A colored border (blue or yellow) around the stimuli was used to further cue participants about which perspective to employ. Block order was pseudo-randomized across participants. Painful and non-painful scenarios were randomized within each block. Post-scan debriefings were conducted to make sure that subjects did follow the perspective-taking instructions.

### MRI acquisition

Scanning was conducted on a 1.5 Tesla Siemens Magnetom Avanto mobile unit equipped with advanced SQ gradients and a twelve element head coil. Functional images were collected using an EPI gradient-echo pulse sequence with TR/TE = 2000/39 ms, flip angle = 90°, field of view = 240 × 240 mm, matrix = 64 × 64 cm, in-plane resolution = 3.4 × 3.4 mm, slice thickness = 5 mm, and 30 slices, full-brain coverage. Task presentation was implemented using the commercial software package E-Prime (Psychology Software Tools, Inc., Pittsburgh PA).

High-resolution T1-weighted structural MRI scans were acquired using a multiecho MPRAGE pulse sequence (repetition time = 2530 ms, echo times = 1.64 ms, 3.50 ms, 5.36 ms, 7.22 ms, inversion time = 1100 ms, flip angle = 7°, slice thickness = 1.3 mm, matrix size = 256 × 256) yielding 128 sagittal slices with an in-plane resolution of 1.0 × 1.0 mm.

### Image processing and analysis

Functional images were processed with SPM8 (Wellcome Department of Imaging Neuroscience, London, UK) in Matlab (Mathworks Inc., Sherborn, MA, USA). For each participant, functional data were realigned to the first image acquisition of the series and re-sampled to a voxel size of 2 × 2 × 2 mm3. Structural T1 images were co-registered to the mean functional image and segmented using the “New Segment” routine. A group-level structural template and individual flow fields were created using DARTEL, and the flow fields were in turn were used to spatially normalize functional images to standard MNI space. Data were smoothed with an 8 mm full-width at half maximum (FWHM) isotropic Gaussian kernel. Three participants were eliminated from further analysis due to issues related to movement or image quality, leaving *N* = 121 (*n* = 40, 47, 37 for low, intermediate, and high psychopathy, respectively).

Statistics were calculated at the first level using the general linear model. The design matrix included three regressors for each stimulus category (detailed above), representing the event onsets and their time and dispersion derivatives. Movement parameters from the realignment output were included as regressors of no interest. All participants were entered into a second-level pooled analysis, and full brain activations were thresholded voxelwise at *p* < 0.001 and with an extent threshold based on Gaussian random fields set to control the whole-brain family-wise error rate (FWE) at *p* < 0.05.

Second-level analyses were conducted by comparing the extremes of the sample distribution of PCL-R scores, and then as a continuous regressor using the entire sample. Participants with PCL-R total score at or above 30 were selected for the psychopathy group, while participants scoring at 20 or below comprised the incarcerated control group. For these analyses, regions of interest (ROIs) were defined using the MarsBar ROI toolbox. We focused on brain regions that were of maximal importance to the hypotheses under investigation, informed by the existing literature on empathy for pain in particular from a meta-analysis of 32 fMRI studies of empathy for pain (Lamm et al., 2011). MNI coordinates were selected from a previous fMRI study of empathy for pain in 80 male incarcerated participants (Decety et al., 2013). That study employed the same 1.5 mobile MRI scanner, and exposed the participants (from a different North American prison) to visual stimuli depicting bodily physical pain and videos of facial expressions of pain. ROI data are reported for significant contrast image peaks within 10 mm of these *a priori* coordinates (FWE-corrected *p* < 0.05). Beyond existing literature on the processing of empathy-inducing stimuli in healthy populations, there may be additional cortical or subcortical brain regions that contribute to abnormal processing of these regions in psychopathy. For instance, the ventral striatum has been found to be over-reactive in adolescents with conduct disorder as well as sexual sadists (Decety et al., 2009; Harenski et al., 2012). Therefore, coordinates for the ventral striatum were selected from a recent meta-analysis of fMRI studies (Diekhof et al., 2012).

To explore the extent to which results found in the groupwise analysis are driven by PCL-R Factor 1, Factor 2, or both, the regions reported above were tested for significant correlation with PCL-R factor scores. Corresponding *t*-values for sub-factor covariates within 10 mm of the ROIs above, if significant, were reported for each factor and task.

### Functional connectivity

Effective connectivity using psychophysiological interaction (PPI, Gitelman et al., 2003) was used to examine the effective connectivity from the anterior insula during imagine-first and imagine-third perspective taking conditions. The right anterior insula was selected because of its role in affective processing and attention. This polysensory region is considered as the integral hub of a salience network, which assists target brain regions in the generation of appropriate behavioral responses to salient stimuli (Menon and Uddin, 2010). Under the hypothesis that high psychopathy may result from a systemic brain deficit which is reflected in abnormal functional-connectivity patterns while imagining pain, we compared effective connectivity in imagine-self perspective and imagine-other perspective conditions between low- and high-psychopathy groups. Because of the importance of the amygdala reactivity (or the lack thereof) in psychopathy, we also ran a similar PPI analysis seeded in the right amygdala.

The time series of the first eigenvariates of the BOLD signal were temporally filtered, mean corrected, and deconvolved to generate the time series of the neuronal signal for the source region—the insula—as the physiological variable in the PPI. The psychological variable represented the time course of the contrast between painful and non-painful trials. An additional regressor represented the interaction of the psychological and physiological factors. These regressors were convolved with the canonical HRF and entered into the regression model. The interaction term in the resulting SPM showed areas with selective connectivity to the insula across the psychological contrast of pain vs. no pain. The PPI analysis was performed for each subject, and the resulting images of contrast estimates were entered into a random-effects group analysis. Second-level analysis results are reported at a voxelwise statistical cutoff of *p* < 0.001 and a spatial extent threshold of *k* > 10 voxels.

## Results

The entire sample of 121 participants (regardless of their psychopathy level) showed significant neuro-hemodynamic increase in the network of regions involved in the actual experience of physical pain under the imagine-self trials (*k* > 10, *p* < 0.05, FWE corrected). This network includes the anterior insula (aINS), anterior midcingulate cortex (aMCC), supplementary motor area (SMA), inferior frontal gyrus (IFG), dorsomedial prefrontal cortex (dmPFC), mPFC, and somatosensory cortex, in both hemispheres (Table 1). In addition, signal change was detected in the left striatum and right amygdala.

**Table 1:** **Imagine-self perspective**.

**Region of interest**		**MNI coordinates**			**Peak *T***
		***x***	***y***	***z***	
L	Anterior insula	−34	20	2	6.59
R	Anterior insula	44	14	0	5.28
L	Supramarginal gyrus	−58	−28	32	6.81
R	Supramarginal gyrus	58	−24	34	6.86
L	Supplementary motor area	−4	12	60	6.38
R	Supplementary motor area	6	10	60	6.35
L	Anterior midcingulate cortex	−6	20	38	6.12
R	Anterior midcingulate cortex	4	18	40	5.29
L	Dorsomedial prefrontal cortex	−8	54	14	5.87
R	Dorsomedial prefrontal cortex	4	56	18	5.29
R	Lateral orbitofrontal cortex	44	30	−4	5.69
L	Inferior frontal gyrus	−38	28	4	6.10
R	Inferior frontal gyrus	54	12	8	5.52
L	Inferior parietal lobule	−44	−54	38	4.73
R	Inferior temporal gyrus	46	−66	−12	5.43
R	Amygdala	20	−4	−14	3.72^*^

Pooled group results for all participants (n = 121).

All clusters are significant at FWE-corrected p < 0.05 (cutoff, t = 4.72), except those marked with a star, which are significant at uncorrected p < 0.0001. L, left hemisphere; R, right hemisphere.

When participants adopted the imagine-other perspective, a similar network was implicated, except for the right amygdala (Table 2). The only additional regions activated were the pSTS and mPFC in the right hemisphere. When imagine-other perspective was contrasted with imagine-self perspective, bilateral activation was detected in the superior parietal cortex (−23, −52, 60 and 27, −44, 59), superior frontal gyrus (−21, −7, 52 and 26, −8, 52), and dorsal striatum (−6, 4, 12 and 9, 4, 11). No significant signal increase was detected for the reverse contrast.

**Table 2:** **Imagine-other perspective**.

**Region of interest**		**MNI coordinates**			**Peak *T***
		***x***	***y***	***z***	
L	Anterior insula	−46	6	−6	7.66
R	Anterior insula	34	28	8	5.45
L	Supramarginal gyrus	−56	−36	36	7.21
R	Supramarginal gyrus	58	−28	28	5.62
L	Supplementary motor area	−4	12	58	5.34
R	Supplementary motor area	6	10	58	6.41
L	Anterior cingulate cortex	−4	24	26	5.67
L	Anterior midcingulate cortex	−6	14	38	6.43
R	Anterior midcingulate cortex	0	−10	34	3.93^*^
L	Dorsolateral prefrontal cortex	−42	40	10	7.07
R	Dorsolateral prefrontal cortex	48	30	0	5.52
L	Dorsomedial prefrontal cortex	−8	56	26	4.72
R	Ventromedial prefrontal cortex	8	54	2	4.04^*^
L	Inferior frontal gyrus	−52	8	6	10.16
R	Inferior frontal gyrus	50	12	4	7.24
L	Post. Superior temporal sulcus	−48	−44	10	3.54^*^
R	Post. Superior temporal sulcus	50	−36	2	5.03
L	Inferior parietal lobule	−44	−34	34	5.17
L	Dorsal striatum	−12	0	4	5.79

Pooled group results for all participants (n = 121). All clusters are significant at FWE-corrected p < 0.05 (cutoff, t = 4.72), except those marked with a star, which are significant at uncorrected p < 0.0001.

## Region of interest analyses

**Figure 1:**
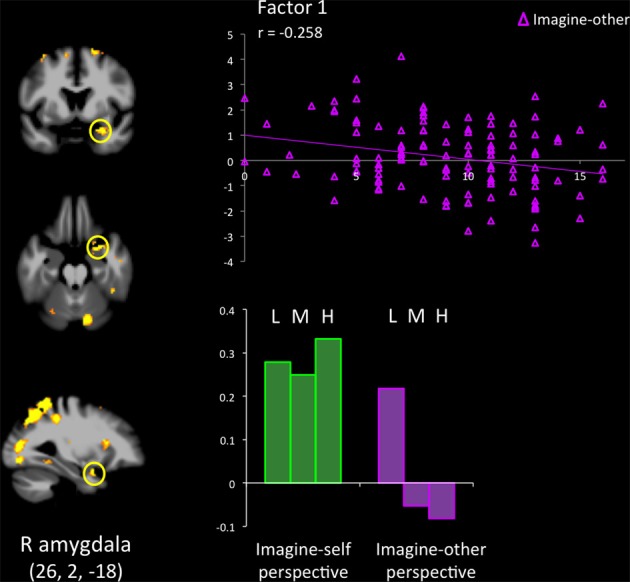
**Response in the right amygdala across groups of low (L), medium (M), and high (H) psychopathy (on total PCL-R scores) participants, when they adopted an imagine-self and an imagine-other affective perspective while viewing bodily injuries.** Groupwise effects (bars at the bottom of the figure) are expanded to show the contribution of continuous PCL-R subscores on Factor 1, which encompasses the emotional/interpersonal features of psychopathy.

Results from the ROI analyses are presented in Table 3. When participants with low scores on the PCL-R were compared with individuals scoring high on the PCL-R, the mPFC (−12, 52, 8) was activated during imagine-self perspective. A cluster of significant hemodynamic increase was found in the OFC. The opposite contrast (high psychopathy > low psychopathy) showed increased signal in the aMCC, SMA, right aINS, IFG, and right pSTS/TPJ. All participants showed significant response in the right amygdala during imagine-self perspective (Figure 1).

**Table 3:** **Groupwise results and factor sub-score covariates for imagine-self and imagine-other perspectives**.

**Region of interest**		**MNI coordinates**			**Peak *T***	**Factor 1**			**Peak *T***	**Factor 2**			**Peak *T***
		***x***	***y***	***z***		***x***	***y***	***z***		***x***	***y***	***z***	
**IMAGINE-SELF PERSPECTIVE**													
Controls > Psychopaths													
R	Orbitofrontal cortex	14	58	−2	3.28				*n.s*.	24	54	2	−2.15
L	dlPFC	−12	52	8	3.40	−16	54	6	−3.12	−14	52	4	−2.44
L	Periaqueductal gray	0	−28	−14	3.23				*n.s*.	−4	−24	−14	−3.01
Psychopaths > Controls													
R	Inferior frontal gyrus	50	26	8	2.65	48	26	10	2.99	52	24	8	2.83
L	Anterior midcingulate cortex	−4	8	34	2.82	−2	8	30	3.31				*n.s*.
R	Anterior midcingulate cortex	4	10	32	3.01	4	10	30	2.93	6	6	32	3.38
L	Supplementary motor area	−10	2	50	2.49	−6	2	54	2.37	−8	6	44	3.18
R	Anterior insula	38	20	12	2.74	32	14	4	2.03	34	20	8	2.73
R	pSTS	44	−48	14	2.41	46	−48	16	2.61	44	−50	18	3.44
**IMAGINE-OTHER PERSPECTIVE**													
Controls > Psychopaths													
R	Inferior frontal gyrus	44	26	2	2.25	40	30	2	−2.62	38	28	10	−2.59
R	Anterior midcingulate cortex	6	18	34	2.21				*n.s*.	8	16	34	−2.44
R	mPFC	16	32	12	3.58	16	32	12	−3.88	12	40	14	−2.67
L	Anterior insula	−44	14	4	2.25	−44	14	4	−2.48	−44	12	0	−2.21
R	Anterior insula	34	30	4	3.07	42	14	2	−2.54	42	10	2	−2.25
L	Supplementary motor area	−6	16	54	3.04	−8	20	54	−2.3	−8	20	54	−4.14
R	Supplementary motor area	8	24	46	2.69	6	26	46	2.13	4	18	52	−2.22
R	pSTS	50	−52	22	3.09	50	−52	20	−3.04	52	−50	16	−2.23
R	Inferior parietal lobule	44	−32	22	2.99	42	−32	22	−3.14	48	−32	26	−3.13
L	Inferior parietal lobule	−48	−36	22	2.81	−44	−38	22	−3.19	−46	−38	22	−3.24
R	Putamen	30	8	2	3.5	30	8	0	−2.7	30	8	2	−2.77
L	Putamen	−14	10	−2	2.48	−12	8	0	−2.56				*n.s*.
Psychopaths > Controls													
R	dlPFC	28	48	14	3.28	30	48	12	2.33	26	50	14	3.60
L	Inferior temporal gyrus	−50	−38	−18	2.62	−52	−40	−16	3.52	−52	−40	−14	3.55
R	Ventral striatum	10	16	−6	3.55	10	16	−6	3.18	12	16	−4	3.84

Negative and positive peak T-values represent negative and positive relations, respectively. L, left hemisphere; R, right hemisphere. dlPFC, dorsolateral prefrontal cortex; mPFC, medial prefrontal cortex; pSTS, posterior superior temporal sulcus.

During the imagine-other perspective, individuals with low scores on the PCL-R compared with individuals with high scores on the PCL-R, showed greater signal change in the SMA, right mPFC, intraparietal sulcus, precentral gyrus, and parahippocampal gyrus/amygdala, pSTS, dorsal aINS and dorsal ACC. In participants with high scores on the PCL-R, the imagine-other perspective was associated with greater activation in the dlPFC and ventral striatum (*p* < 0.001), when compared to low-scoring incarcerated controls.

## Correlations between PCL-R scores and ROIs

**Figure 2:**
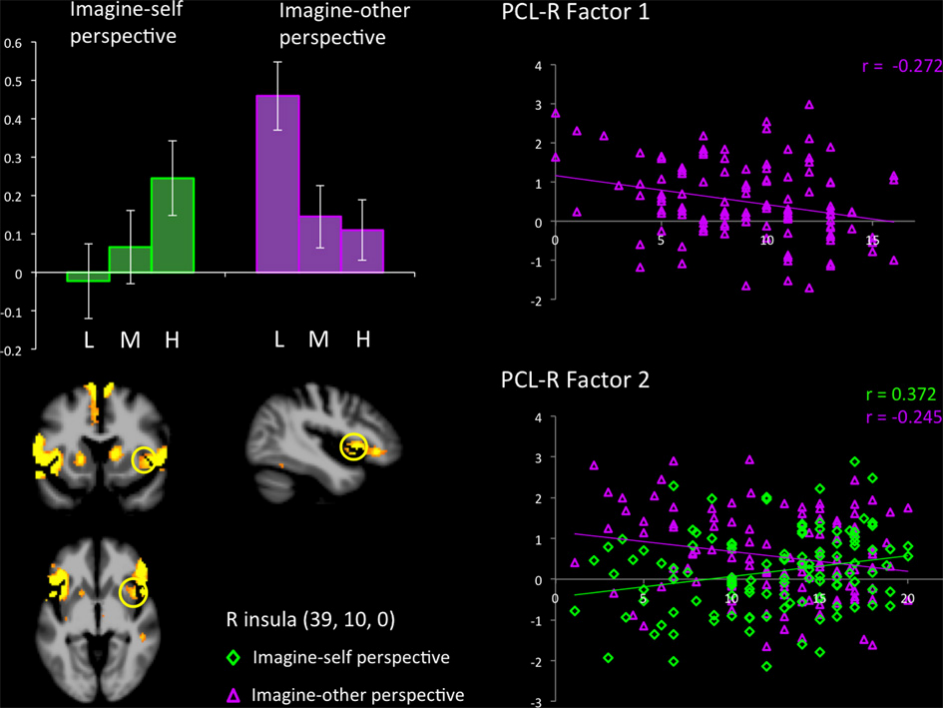
**Response in the right anterior insula across groups (L, low; M, medium; H, high on total PCL-R scores) during imagine-self and imagine-other perspectives in participants viewing bodily injuries.** Groupwise effects seen in (bar graph) are expanded to show the contribution of Factors 1 and 2 from PCL-R subscores.

**Figure 3:**
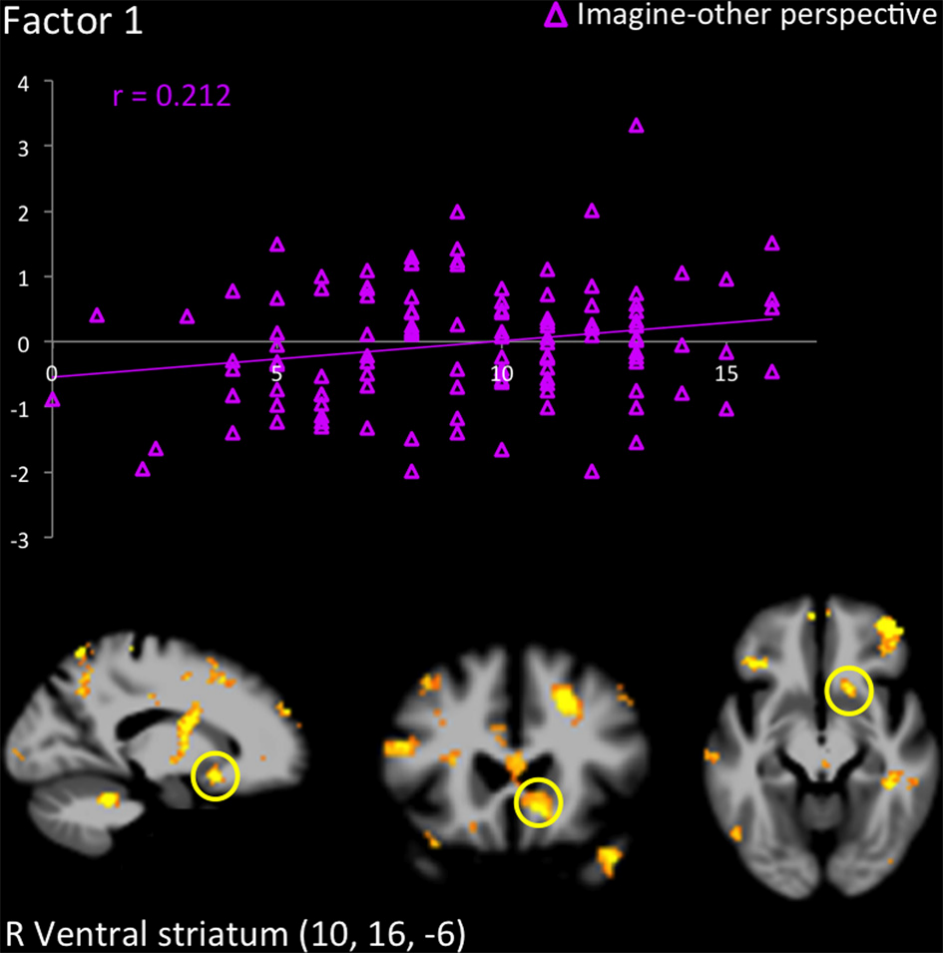
**Response in the right ventral striatum in participants scoring high on the PCL-R (≥30) when they imagined another person in pain, and correlation with scores on Factor 1**.

The hemodynamic response in the aINS was significantly greater in individuals scoring high on psychopathy (total PCL-R score) during imagine-self perspective, and the reverse was found for imagine-other perspective (Figure 2). Factor 2 positively correlated with the activity in aINS during imagine-self perspective (*r* = 0.372, *p* = 0.016), whereas it negatively correlated with aINS activity during imagine-other perspective (*r* = −0.254, *p* = 0.01). Factor 1 was negatively correlated with response in aINS during third-person perspective (*r* = −0.272, *p* = 0.01). Activity in the dmPFC was negatively associated with both Factor 1 (*r* = −0.24, *p* < 0.01) and Factor 2 (*r* = −0.237, *p* = 0.01) during imagine-self perspective. The hemodynamic response in the dlPFC was positively correlated with both Factor 1 (*r* = 0.288, *p* < 0.01) and Factor 2 (*r* = 0.274, *p* < 0.01) during imagine-other perspective. The response in the ventral striatum during imagine-other perspective significantly correlated with scores on Factor 1 (*r* = 0.212, *p* < 0.02, see Figure 3). Finally, response in the right amygdala (26, 2, −18) showed a negative correlation with Factor 1 (*r* = −0.258, *p* = 0.04) during imagine-other perspective. No significant correlation was found in imagine-self perspective with either Factors 1 and 2. See Table 3 for a complete list of results.

## Effective connectivity analyses

**Figure 4:**
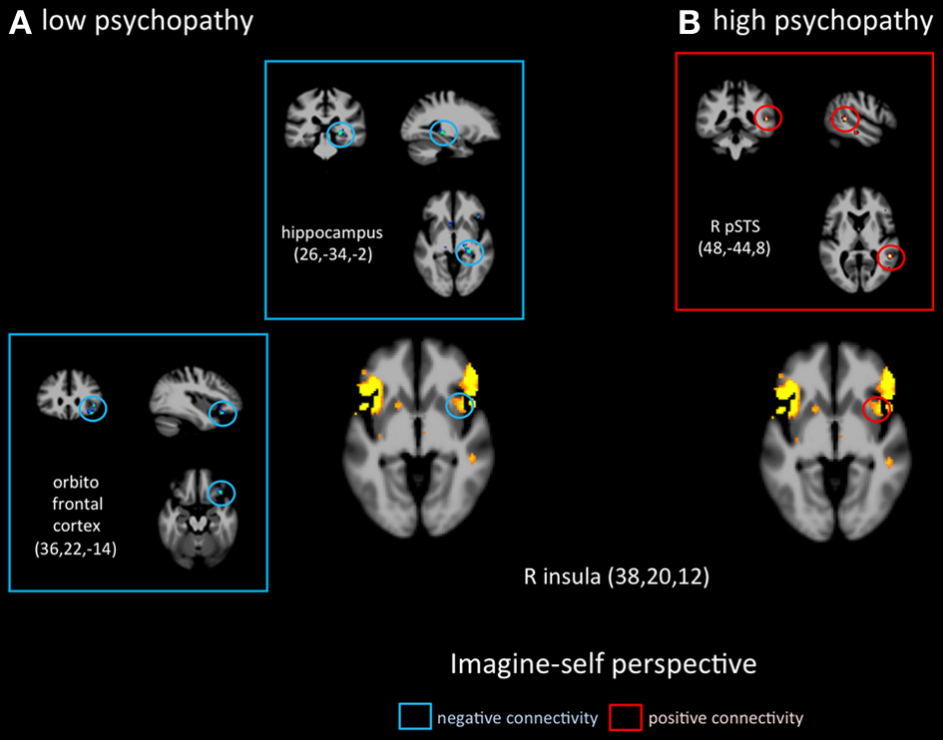
**Functional connectivity analyses, seeded in the anterior insula in participants with the lowest scores on the PCL-R (≤20) and participants with the highest scores on the PCL-R (≥30) during imagine-self perspective**.

**Figure 5:**
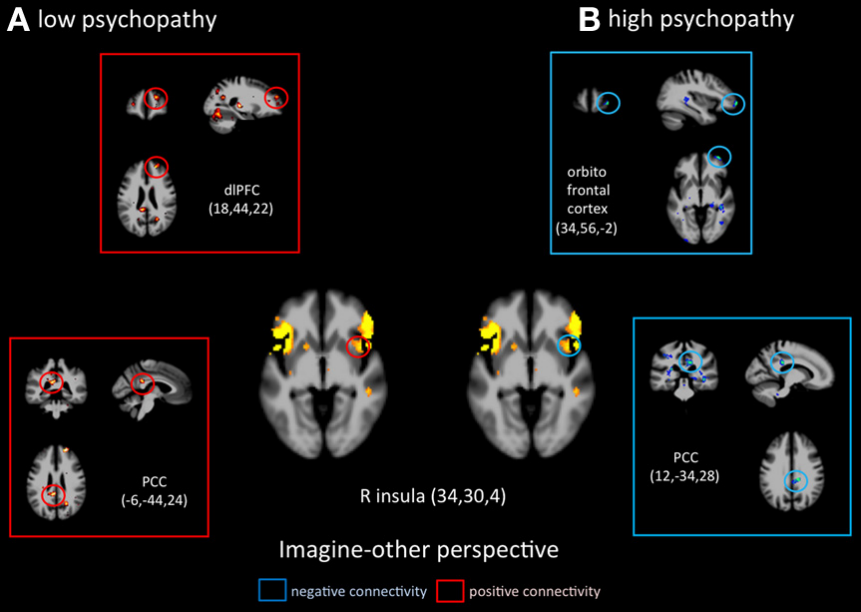
**Functional connectivity analyses, seeded in the anterior insula in participants with the lowest scores on the PCL-R and participants with the highest scores on the PCL-R (>30) during imagine-other perspective**.

Functional connectivity analyses seeded in the anterior insula revealed distinct patterns in functional coupling between the low- and high-psychopathy groups. During imagine-self perspective, individuals scoring low on the PCL-R showed a negative connectivity between the aINS and the hippocampus and the OFC (Figure 4). In the high psychopathy group, there was only significant functional connectivity between the aINS and the right pSTS. During imagine-other perspective, low-psychopathy participants had significant effective connectivity between the aINS and posterior cingulate cortex and dlPFC (Figure 5). In high-scoring participants, negative connectivity was found between aINS and the right OFC and posterior cingulate cortex.

**Figure 6:**
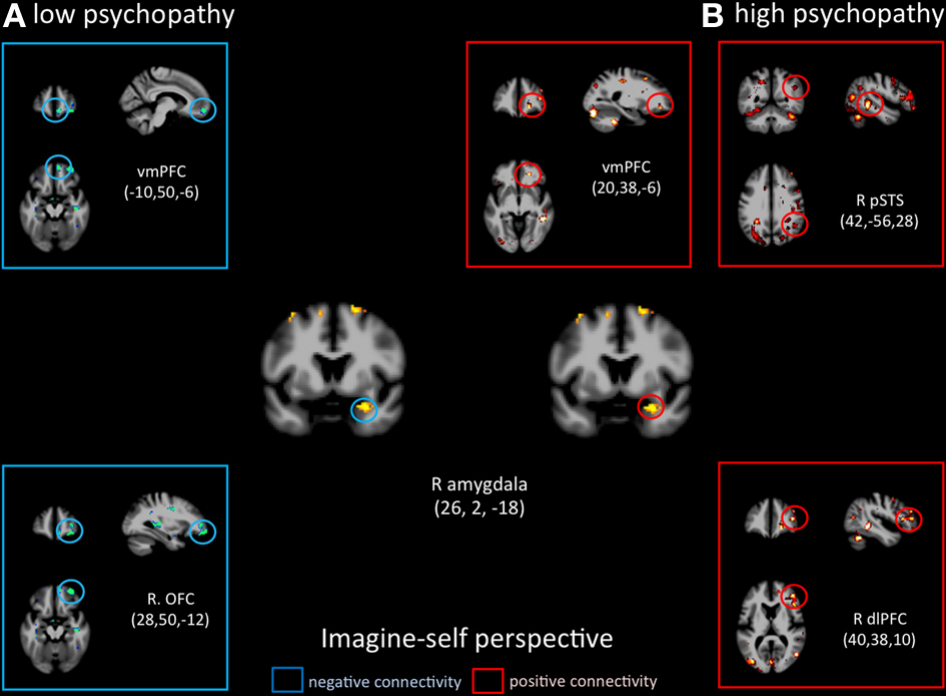
**Functional connectivity analyses, seeded in the right amygdala in participants with the lowest scores on the PCL-R (≤20) and participants with the highest scores on the PCL-R (≥30) during imagine-self perspective**.

**Figure 7:**
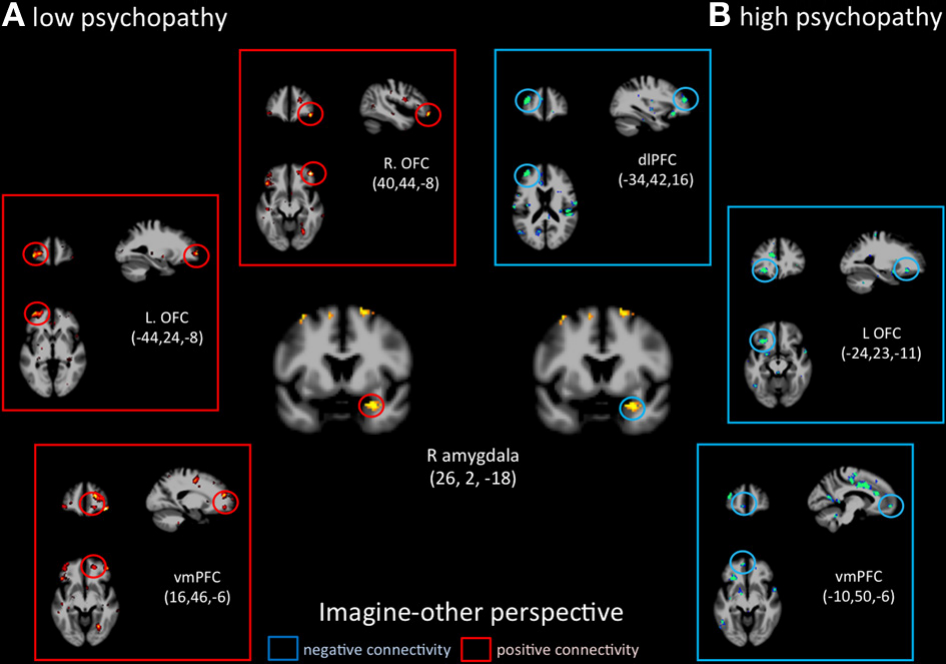
**Functional connectivity analyses, seeded in the right amygdala in participants with the lowest scores on the PCL-R and participants with the highest scores on the PCL-R (>30) during imagine-other perspective**.

Functional connectivity analyses seeded in the right amygdala showed distinct patterns of co-variations depending on the perspective adopted in controls vs. psychopaths. During imagine-self perspective, controls exhibited a significant negative coupling between the amygdala and ventral and mPFC, while participants with high scores on the PCL-R showed a positive coupling with the pSTS/TPJ, ventral and mPFC, and dlPFC (Figure 6). During imagine-other perspective, the reverse pattern of functional connectivity was observed. Low psychopathy was associated with greater positive coupling with the OFC, whereas the high psychopathy showed a negative coupling with the OFC and dlPFC (Figure 7).

## Discussion

Perspective taking while observing or imagining other's feelings has been described as an empathic attentional set that facilitates other-oriented emotional and motivational responses congruent with the perceived welfare of that person (Van Lange, 2008; Batson, 2012). To examine the extent to which affective reactions can be evoked or modulated by perspective taking in individuals with psychopathy, incarcerated participants with different levels on the PCL-R were scanned while viewing stimuli depicting bodily injuries and instructed to imagine these situations as either happening to themselves or to someone else.

At the group level, collapsed across the PCL-R scores (*n* = 121), both conditions of imagine-self and imagine-other in pain were associated with signal increase in brain regions implicated in the perception of pain and distress, when viewing body parts suffering injuries or facial expressions of pain (Jackson et al., 2006; Lamm et al., 2007, 2011; Decety and Porges, 2011; Bruneau et al., 2012). In healthy participants, activity in this network, which includes the aINS, thalamus, aMCC, IFG, and somatosensory cortex, has been interpreted as a form of somatosensory resonance, or shared neural representations with the pain of others, providing an implicit intersubjective affective knowledge (Decety and Jackson, 2004; Singer and Decety, 2011; Zaki and Ochsner, 2012). However, these vicariously instigated activations of the so-called “pain matrix” are not specific to the sensory qualities of pain, but instead are associated with more general survival mechanisms such as aversion and withdrawal when exposed to danger and threat (Benuzzi et al., 2008; Decety, 2010). In fact, based on a systematic review of electroencephalographic and functional MRI studies that examined neural response triggered by nociceptive stimuli, activity of this cortical network seems to reflect a system involved in detecting, processing, and reacting to the occurrence of salient sensory events regardless of the sensory channel through which these events are conveyed (Legrain et al., 2011).

Interestingly and quite surprisingly, the hemodynamic response in aINS and aMCC, regions considered as pivotal in the affective component of empathy, was highest in high psychopaths during imagine-self perspective, replicating the results of a recent study of pain empathy in criminal psychopaths that reported greater activation in the insula, which was positively correlated with scores on both PCL-R factors 1 and 2 (Decety et al., 2013) (Figure 2). The aINS and aMCC are the two regions that have been most reliably activated in fMRI studies of pain empathy with healthy subjects (Valentini, 2010; Lamm et al., 2011). This finding does not support the view that psychopaths do not resonate when exposed aversive stimuli such as pain, or at least they are not totally blunted when they take a first-person perspective. This finding also raises an interesting question: whether or not sensorimotor resonance (underpinned by the mirror neuron system involved in perception-action coupling) is the mechanism that facilitates emotion contagion and empathic arousal. Psychopaths are characterized by a lack of affective empathy, but there is little evidence that they show a deficit in sensorimotor resonance (Blair, 2011; Decety, 2011b). For instance, a transcranial magnetic stimulation study demonstrated increased sensorimotor resonance to painful hand-pricking videos in college students scoring high on the psychopathic personality inventory (PPI), as compared to students who score low on the PPI (Fecteau et al., 2008). Juvenile incarcerated psychopaths showed greater sensorimotor resonance as measured by EEG and suppression of the *mu* rhythm when they viewed visual stimuli depicting people being physically injured, despite a lack of affective arousal to the same stimuli as measured by the N120 ERP component (Cheng et al., 2012). Children with aggressive conduct disorder and psychopathic tendencies and incarcerated psychopaths exhibit typical (Marsh et al., 2013) or even stronger activation in the somatosensory cortex than control participants when they watched scenarios depicting people in pain (Decety et al., 2009, 2013), all of which does not suggest an impairment in somatosensory responses to others' pain. Our finding that participants scoring high on psychopathy activate the pain network during imagine-self perspective fits well with studies showing that individuals with psychopathy may up-regulate emotional (at least for fear) processing when attention to salient stimuli is particularly engaged (Newman and Lorenz, 2003), and this may be the case for pain.

Furthermore, and as expected, the lower the participants scored on Factors 1 and 2 of the PCL-R, the higher the activity in the aINS during imaging-other perspective. This indicates that more vicarious experience was elicited in control participants when they imagined another in pain, and the opposite pattern (low activation in the aINS) was found in participants who scored high on psychopathy. In addition, functional connectivity analyses, seeded in the right aINS during imagine-self perspective negatively co-varied with activation in the hippocampal gyrus and OFC in control participants (low on psychopathy), and was positively coupled with the right pSTS region in psychopaths. During imagine-other perspective, the aINS positively covaried with activity in the right dlPFC and PCC in controls, and negatively with the OFC and PCC in high psychopaths. Altogether, the hemodynamic response in the aINS shows distinct profiles of activation depending on whether participants adopted an imagine-self or imagine-other perspective taking. These results from the imagine-other perspective condition support two recent functional neuroimaging studies in children with conduct disorder (Lockwood et al., 2013; Marsh et al., 2013). Both studies reported a reduced response in the aINS and ACC when the children viewed pictures of others in pain. Furthermore, a negative association between callous traits and the aINS/ACC was found. The fact that individuals with high scores on the PCL-R showed a reduced response when imagining the pain of another suggests a specific deficit in affective processing in a region considered as a critical hub to integrate salient stimuli and events with visceral and autonomic information (Menon and Uddin, 2010).

Signal change in the right amygdala was detected during imagine-self perspective in all participants, and during imagine-other perspective in controls. The hemodynamic response in the amygdala was inversely correlated with individual scores on PCL-R Factor 1 during imagine-other perspective. This is in line with most neuroimaging studies of psychopathy that documented reduced amygdala response to fearful and aversive stimuli (Marsh and Blair, 2008; Harenski et al., 2009). This finding is consistent with the notion that psychopaths lack the ability to be responsive to, or aroused by distress cues, and therefore are not sensitive to signs of vulnerability. A recent fMRI study in youths with psychopathic traits also reported reduction in the amygdala and insula when they imagined physical injuries to others, but not their own pain (Marsh et al., 2013).

It is very interesting to note that imagine-self perspective was associated with activity in the amygdala in psychopaths when they focus on their own affective reaction. While most studies report a reduced response in the amygdala in psychopaths, an fMRI study conducted on a small number psychopaths and controls found increased activation in the right amygdala in the psychopath group with respect to controls when viewing negative IAPS pictures (Müller et al., 2003), indicating that the role of the amygdala in psychopathy may not be straightforward, nor its lateralization. A meta-analysis of 67 neuroimaging studies reported that the lateralization of activation in the amygdala was explained by differences in temporal dynamics and/or habituation rates, namely a short-duration response in the right amygdala and a more sustained one in the left (Sergerie et al., 2008). It is however difficult to interpret the amygdala activation during imagine-self perspective further without a more fine-grain analysis of amygdala sub-nuclei and their anatomical connectivity, which helps determine their function (Saygin et al., 2011). With this caveat in mind, it is important to note that functional connectivity analyses, seeded in the right amygdala, demonstrated very different patterns of connectivity depending on the perspective taking strategy (imagine-self vs. imagine-other) and participants (low vs. high psychopaths). The response in the right amygdala was negatively coupled with activity in the OFC in controls and positively correlated with the OFC and dlPFC and pSTS in high psychopathy during imagine-self perspective (Figure 3). The exact reverse functional connectivity was detected during imagine-other perspective (Figure 4). This finding specifically points to amygdala–OFC interactions as being an important neural mechanism that underlies the outcome of perspective taking in psychopathy. It seems to indicate that during imagine-self perspective, individuals with psychopathy elicit amygdala-OFC coupling but fail to do so during imagine-other perspective. Such a failure to recruit the OFC during third-person perspective taking supports the dysfunction of this neural pathway in response to distress cues of others in psychopaths. It has been argued that the integrated functioning of this circuit enables the basics of care-based morality, and that dysfunction within these regions in psychopathy means that reinforcement-based decision making, including moral decision making, and care base morality is impaired (Blair, 2007; Shamay-Tsoory et al., 2010; Marsh et al., 2011). One theory of the origin of empathic deficits in psychopathy is the failure during development to form stimulus-reinforcement associations connecting harmful or aggressive actions with the pain and distress of others (Kiehl, 2006; Glenn and Raine, 2009). It is worth mentioning that psychopathic traits are not exclusively associated with amygdala hyporeactivity. A study that included 200 young adults with self-reported psychopathy assessment found that amygdala reactivity to fearful facial expressions is negatively associated with the interpersonal facet of psychopathy, whereas reactivity to angry expressions is positively associated with the lifestyle facet (Carré et al., 2013).

Finally, the increase of activity in the ventral striatum during imagine-other perspective in psychopaths, which was predicted by their scores on Factor 1 of the PCL-R, is an intriguing finding. This could suggest that psychopaths not only experience blunted vicariously arousal to others' pain and reduced feelings of concern when adopting their perspective, but they may in fact find the distress of others pleasurable or positively arousing. The ventral striatum is selectively recruited during reward anticipation in healthy participants (Diekhof et al., 2012 for a meta-analysis). In adolescents with conduct disorder and psychopathic tendencies, an fMRI study found activation of the ventral striatum during the perception of pain in others (Decety et al., 2009). In healthy subjects, the ventral striatum has been associated with experiencing pleasure at others' misfortune (e.g., Dvash et al., 2010; Cikara et al., 2011). It has been suggested that neurons in the ventral striatum have access to central representations of reward and thereby participate in the processing of information underlying the motivational control of goal-directed behavior (Schultz et al., 1992). Activation of the ventral striatum while imaging another in physical pain was correlated with PCL-R Factor 1, and not Factor 2. Abnormalities in the ventral and dorsal striatum are considered to play a key role in the etiology of psychopathic traits (Buckholtz et al., 2010; Carré et al., 2013)

## Conclusion

There is general consensus among theorists that the ability to adopt and entertain the psychological perspective of others has a number of important consequences, including empathic concern (e.g., Blair, 2007; Batson, 2009; Decety and Svetlova, 2012). Adopting the perspective of another is a powerful way to place oneself in the situation or emotional state of that person (Batson, 2011). Our results demonstrate that while individuals with psychopathy exhibited a strong response in pain-affective brain regions when taking an imagine-self perspective, they failed to recruit the neural circuits that are were activated in controls during an imagine-other perspective, and that may contribute to lack of empathic concern. Finally, this atypical pattern of activation and effective connectivity associated with perspective taking manipulations may inform intervention programs in a domain where therapeutic pessimism is more the rule than the exception (Salekin, 2002). Altered connectivity may constitute novel therapeutic targets for interventions. Both cognitive and pharmacotherapy interventions may restore connectivity patterns (Crocker et al., 2013). Imagining oneself in pain or in distress may trigger a stronger affective reaction than imagining what another person would feel, and this could be used with some psychopaths in cognitive-behavior therapies as a kick-starting technique for eliciting emotional tagging of different outcomes of interpersonal situations.

### Conflict of interest statement

The authors declare that the research was conducted in the absence of any commercial or financial relationships that could be construed as a potential conflict of interest.
